# ^99m^Tc internal contaminations measurements among nuclear medicine medical personnel during ventilation – perfusion SPECT lung scans

**DOI:** 10.1007/s00411-021-00905-x

**Published:** 2021-03-22

**Authors:** E. Borkowska, K. Brudecki, M. Kostkiewicz, K. Gorzkiewicz, R. Misiak, E. Nalichowska, J. Miszczyk, T. Mróz

**Affiliations:** 1 Electroradiology Department, Faculty of Health Sciences, Institute of Physiotherapy, Jagiellonian University, Collegium Medicum, Michalowskiego 12, 31-126 Kraków, Poland; 2 Institute of Nuclear Physics, Polish Academy of Sciences, Radzikowskiego 152, 31-342 Kraków, Poland; 3 Heart and Vascular Diseases Department, Faculty of Medicine, Institute of Cardiology, Jagiellonian University, Collegium Medicum, Prądnicka 80, 31-202 Kraków, Poland; 4 Nuclear Medicine Department, John Paul II Hospital, Prądnicka 80, Kraków, Poland; 5 Institute of Physics, Jagiellonian University, Łojasiewicza 11, 30-348 Kraków, Poland

**Keywords:** ^99m^Tc, Internal contamination, Air, Blood, Medical personnel, Dose

## Abstract

This paper presents results of measurements of ^99m^Tc activity concentration in air and nuclear medical personnel blood during ventilation–perfusion SPECT lung scans. ^99m^Tc activity measurements were conducted at the Nuclear Medicine Department, John Paul II Hospital, Krakow. Technicians and nurses who perform examinations were equipped with personal aspirators enabling air sampling to determine the radiation exposure at their workplaces. Measurements allowed to evaluate the concentration of ^99m^Tc in 14 air samples and it ranged from 7800 ± 600 to 10,000 ± 1000 Bq m^−3^ for air samples collected by technicians and from 390 ± 30 to 600 ± 40 Bq m^−3^ for air samples collected by nurses. In addition ^99m^Tc concentrations in blood of medical personnel were determined in 24 samples. For technicians the maximum ^99m^Tc blood concentration levels reached 920 ± 70 Bq L^−1^ and 1300 ± 100 Bq L^−1^. In the case of nurses, the maximum estimated activity concentrations were about ten times lower, namely 71 ± 7 Bq L^−1^ and 39 ± 3 Bq L^−1^. Although the intakes appear to be relatively high, the resulting annual effective doses are about 34 µSv for technicians and only 2 µSv for nurses.

## Introduction

Lung scintigraphy is a medical imaging technique used in diagnosis of respiratory system diseases. It consists of perfusion scintigraphy and ventilation scintigraphy. In the first case, examination focusses on assessment of blood supply to the lung tissue, i.e., blood circulation in supplying vessels. It uses intravenous injection of blood proteins (albumins) previously marked with ^99m^Tc. To study the ability of air to reach all parts of the lungs, ventilation scintigraphy is performed. Radioactive gas (^133^Xe) or aerosols (^99m^Tc-diethylenetriaminepentaacetate—DTPA, Technegas) is inhaled by patients by means of a mouthpiece or mask which covers the mouth and nose. The last part of examination consist of medical imaging with a SPECT (single-photon emission computed tomography) camera which registers gamma radiation emitted by decaying radioisotopes, allowing to identify congestions and constrictions in the respiratory or circulatory systems. Both medical tests—perfusion and ventilation scintigraphy—are commonly performed simultaneously for optimal assessment of patient's health condition.

To perform ventilation scintigraphy, a patient inhales gas containing 400 MBq of ^99m^Tc. Predominantly, atomized DTPA or Technegas (ultrafine dispersion of ^99m^Tc labeled carbon) are used. Previous research projects proved that during inhalation of gas marked with ^99m^Tc, part of its activity leaks into the room air and may pose a risk for medical team that performs tests (Martinez et al. 2019; Brudecki et al. 2019).

In 2018, measurements of ^99m^Tc activity in indoor air at the Nuclear Medicine Department, John Paul II Hospital, (Krakow, Poland) during ventilation–perfusion lung scan treatments were carried out. Determined concentrations ranged from (99 ± 11) Bqm^−3^ to (6088 ± 479) Bqm^−3^. Additionally, medical staff's daily average intakes were estimated to be 4400 Bq and 2450 Bq for technicians female and nurses female, respectively, what resulted in annual effective doses at the level of 1 µSv (Brudecki et al. 2019).

This paper addresses the problem of assessment of internal contaminations with ^99m^Tc among medical staff routinely performing ventilation–perfusion SPECT lung scans. The proposed assessment is based on an innovative system of personal air aspirators. Additionally, the presented research is supplemented with the measurements of ^99m^Tc activity in the blood of medical personnel.

## Materials and methods

### Medical personnel

The investigation was performed among medical staff of the Nuclear Medicine Department, John Paul II Hospital, (Krakow, Poland). Ten employees, namely 3 technicians, 1 physicist, 4 nurses and 2 doctors work in the facility. Annually, they perform about 3,600 diagnostic tests by means of SPECT camera and ^99m^Tc. This number includes heart scintigraphy (2000), kidney scintigraphy (200), bone scintigraphy (1000) and lung ventilation and perfusion scintigraphy (400).

Lung ventilation–perfusion scintigraphy procedure is performed only once a week (on Tuesdays) and four tests are conducted. The department utilizes Technegas generator manufactured by Cyclomedica (Germany). Each lung ventilation and perfusion scintigraphy procedure is performed by a technician and a nurse. The technician is present during inhalation of Technegas and subsequently helps in placing the patient under the SPECT camera. On the other hand, the nurse injects patient intravenously ^99m^Tc in the perfusion part of the procedure.

### Air sampling

**Fig. 1:**
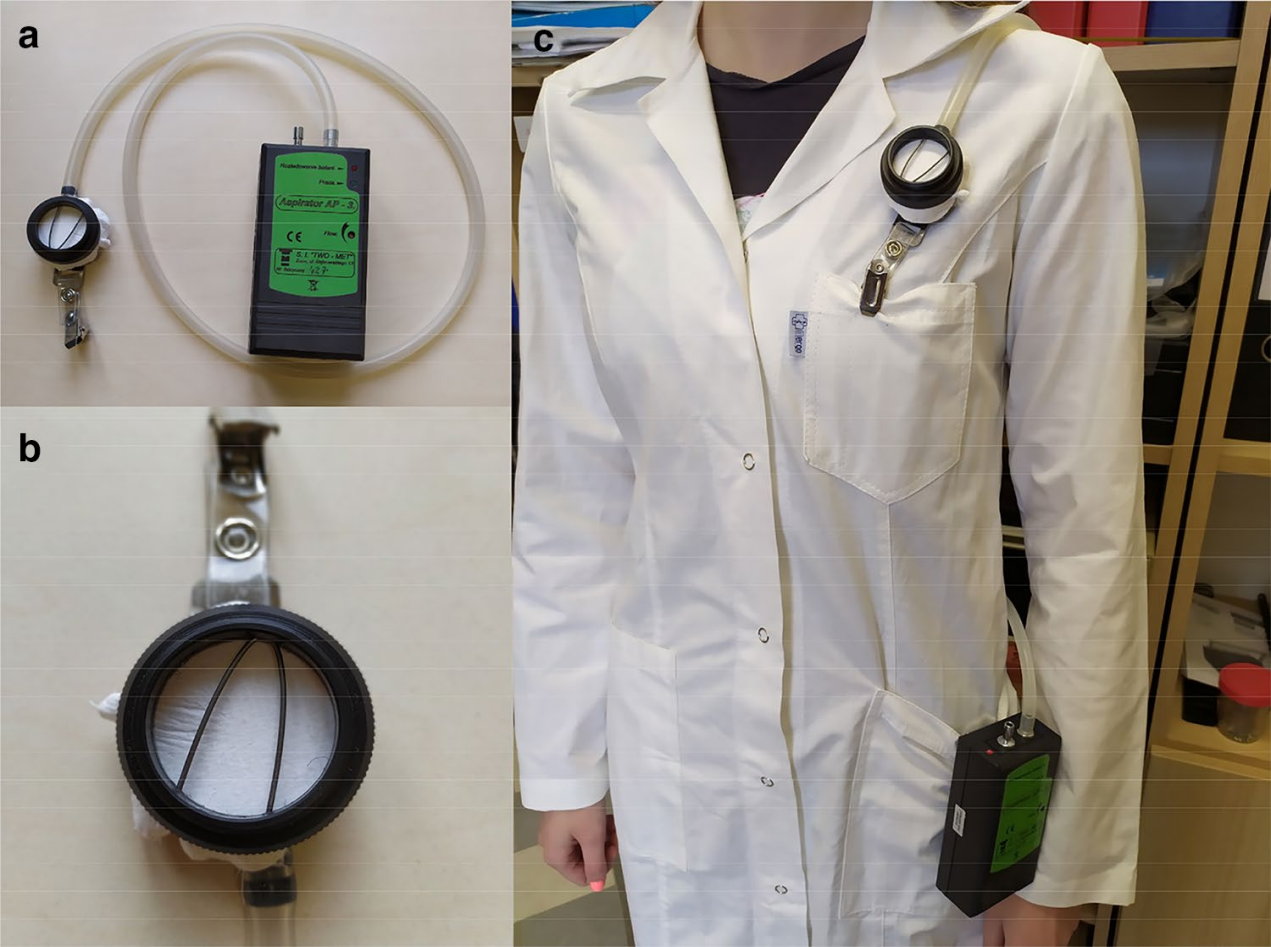
Personal aspirator for air sampling, **a** aspirator, **b** Petryanov filter FPP-15–1.5, **c** aspirator worn by medical personnel

The personnel performing the examinations were equipped with personal aspirators for air sampling to determine exposure at workplaces. AP-8 aspirators, produced by Two-Med, Poland, were used in the research. Aspirators enable air flow in the range from 1 to 4 L min^−1^ with flow stabilization up to 5%. Battery capacity allows for 10 h of continuous operation. Due to the fact that Technegas is supplied to the patient's lungs in the form of an aerosol, the aspirators were equipped with the Petryanov filter FPP-15–1.5 (poly (vinyl chloride)). Figure 1 shows the set used in the presented investigations.

Tests with the use of aspirators were carried out during 6 working days when perfusion–ventilation scans were performed and on 1 day when these procedures were not performed (to determine the background levels). Each time, the technician and the nurse who were performing the examinations were equipped with aspirators. In total, 14 air samples were collected using the described method.

### Blood sampling

Four employees (2 technicians and 2 nurses) took part in the investigation focusing on the determination of ^99m^Tc activity in blood among medical personnel. Blood samples were drawn during 2 working days, when ventilation–perfusion lung scans were performed. From every person, that took part in the experiment, six blood samples, 10 mL each, were collected. The blood samples were drawn before, after two and four ventilation–perfusion SPECT lung scans and subsequently every each hour till end of work. Overall, the activity of ^99m^Tc was determined in 24 blood samples. It is worthwhile to stress that the carried out investigation, especially the blood sampling, is very burdensome for the medical personnel. Therefore, in order not to pose additional threat to both medical staff and patients, only four people were selected for the tests. The study was approved by the Bioethics Committee at the Regional Medical Chamber in Krakow, Poland (decision number 92/KBL/OIL/2020, dated 05.06.2020).

### Gamma measurement

Samples were measured by a low-background gamma ray spectrometer located in the Department of Nuclear Physical Chemistry, Institute of Nuclear Physics Polish Academy of Sciences (IFJ PAN), Krakow, Poland. The spectrometer is equipped with a Broad Energy Germanium (BEGe) detector BE5030 (relative efficiency ≥ 48% 0.6 mm Carbon Composite entrance window and 102 mm diameter of endcap) manufactured by Canberra (USA). This detector offers high efficiency, especially for low energy gamma lines (like 140 keV ^99m^Tc spectrum line). The detector is supplied with 3.5 kV high voltage by Canberra Model 3106D Power Supply (Gorzkiewicz et al. 2019).

Passive shield of the spectrometer consist of several layers, namely (from outside to inside): paraffin, plastic scintillator (playing role of active shielding, did not used in described measurements), 10 cm of standard lead bricks, 2 mm of cadmium, 5 cm of lead casted over 2500 years ago, 1 cm of electrolytic copper and acrylic organic glass (Mietelski et al. 2004). Additionally, in order to lower the concentration of radon daughters, flush of vapours of liquid nitrogen are used to purge spectrometer’s inner chamber (Gorzkiewicz et al. 2019).

The data acquisition was performed by means of a Mutli-channel Pulse Amplitude Analyzer TUKAN 8 k governed by the Tukan 8 k (NCNR, Poland) software used in acquisition, store and analysis of obtained results. The concentration of ^99m^Tc was determined by analysis of 140.5 keV line (intensity 89%).

### Biokinetic modeling

Biokinetic modeling was used to compare the results obtained from measurements of air and blood samples. For this purpose, the technetium biokinetics model (ICRP 2016) was used in connection with the Human Respiratory Tract Model (HRTM) (ICRP 1994,2002). Both models were developed by International Commission on Radiological Protection (ICRP). The SAAM II software (Epsilon group, USA) was used in the modeling. This method has been successfully used many times in previous studies (Li et al. 2008; Brudecki et al. 2014, 2017a, 2017b, 2018a, 2018b, 2019, 2020).

In performed simulations, the rate of Technegas absorption into the blood was assumed as type F (fast) (ICRP 2002). The manufacturer of the Technegas system claims that the generated aerosol particles diameters are in the range of 30–60 nm (Cyclomedica 2007). Therefore, the ^99m^Tc aerosol deposition conditions were adopted for the activity median aerodynamic diameter (AMAD) of the attached aerosols equal 50 nm. Additionally, the breathing rate of medical staff members during work was assumed to correspond to light exercise physical activity.

### Dose estimations

Intakes calculated on the base of average activity of ^99m^Tc in air samples collected by means of aspirators along with dose conversion factors allowed to estimate the effective doses. Dose coefficient equal 1.5 × 10^–11^ Sv Bq^−1^ was used during calculations, which value was taken from ICRP Database of Dose Coefficients: workers and members of the Public ver 3.0. To calculate the annual doses, 200 treatments per year were assumed (50 work days, 4 treatments per day).

## Results and discussion

^99m^Tc activity concentration were determined in 14 air samples. In case of 12 samples collected at the time when the treatments were performed, the measured Tc concentrations were above the detection limits and ranged from 7800 ± 600 to 10,000 ± 1000 Bq m^−3^ for air samples collected by aspirators carried by technicians and from 390 ± 30 to 610 ± 40 Bq m^−3^ in the case of samples collected by aspirators worn by nurses. The two samples collected to estimate background concentrations were below the detection limits. This confirms the assumption that the only procedure that causes contaminations among medical personnel during performed studies were ventilation–perfusion lung scans.

Taking into account the obtained activities, duration of the performed medical examinations (4 h) and assuming a breathing rate of 1.2 m^3^ h^−1^, characteristic for light exercising women (women represent 80% of the medical personnel in the facility, where studies were carried out and only women participated in the presented study), the daily intakes of ^99m^Tc were estimated to be in range from 37,000 ± 3000 to 48,000 ± 5000 Bq with mean value 43,000 ± 4000 Bq for technicians and from 1900 ± 100 to 2900 ± 200 Bq with mean 2400 ± 300 Bq for nurses. During the days when research was conducted, the medical staff members performed four treatments per day, so one treatment may cause an intake at the level 11,000 Bq for technicians and about 600 Bq for nurses. The exact results are presented in Table 1.

**Table 1:** Results of ^99m^Tc concentration measurements in air samples collected using personal air aspirators (*C*) and calculated daily intakes (*I*_d_)

Sample	*C* [Bq m^−3^]	*I*_d_ [Bq]
Day 1		
Technician	8600 ± 600	41,000 ± 3000
Nurse	460 ± 30	2200 ± 200
Day 2		
Technician	7800 ± 600	37,000 ± 3000
Nurse	600 ± 40	2900 ± 200
Day 3		
Technician	9100 ± 700	44,000 ± 3000
Nurse	530 ± 40	2500 ± 200
Day 4		
Technician	10,000 ± 1000	48,000 ± 5000
Nurse	500 ± 40	2400 ± 200
Day 5		
Technician	9200 ± 700	44,000 ± 3000
Nurse	490 ± 40	2400 ± 200
Day 6		
Technician	9500 ± 700	46,000 ± 3000
Nurse	390 ± 30	1900 ± 100
Average		
Technician	9000 ± 800	43,000 ± 4000
Nurse	500 ± 70	2400 ± 300
Day 7 (references level*)		
Technician	< MDC	–
Nurse	< MDC	–

The results are presented with one sigma uncertainty, or in the case of average values standard deviations

*Measurements were carried out when ventilation–perfusion scans were not performed

In the case of nurses, those results are in good agreement with previous studies (Brudecki et al. 2019), focused on measurements of ^99m^Tc air activity during scintigraphy procedures, which allowed to estimate intakes at 2500 Bq. However, for technicians, the obtained results are ten times higher. This may be due to the choice of air sampling site in the previous studies that could be non-representative for technicians as well as operation of the high-efficiency ventilation system disturbing the sampling procedure.

**Fig. 2:**
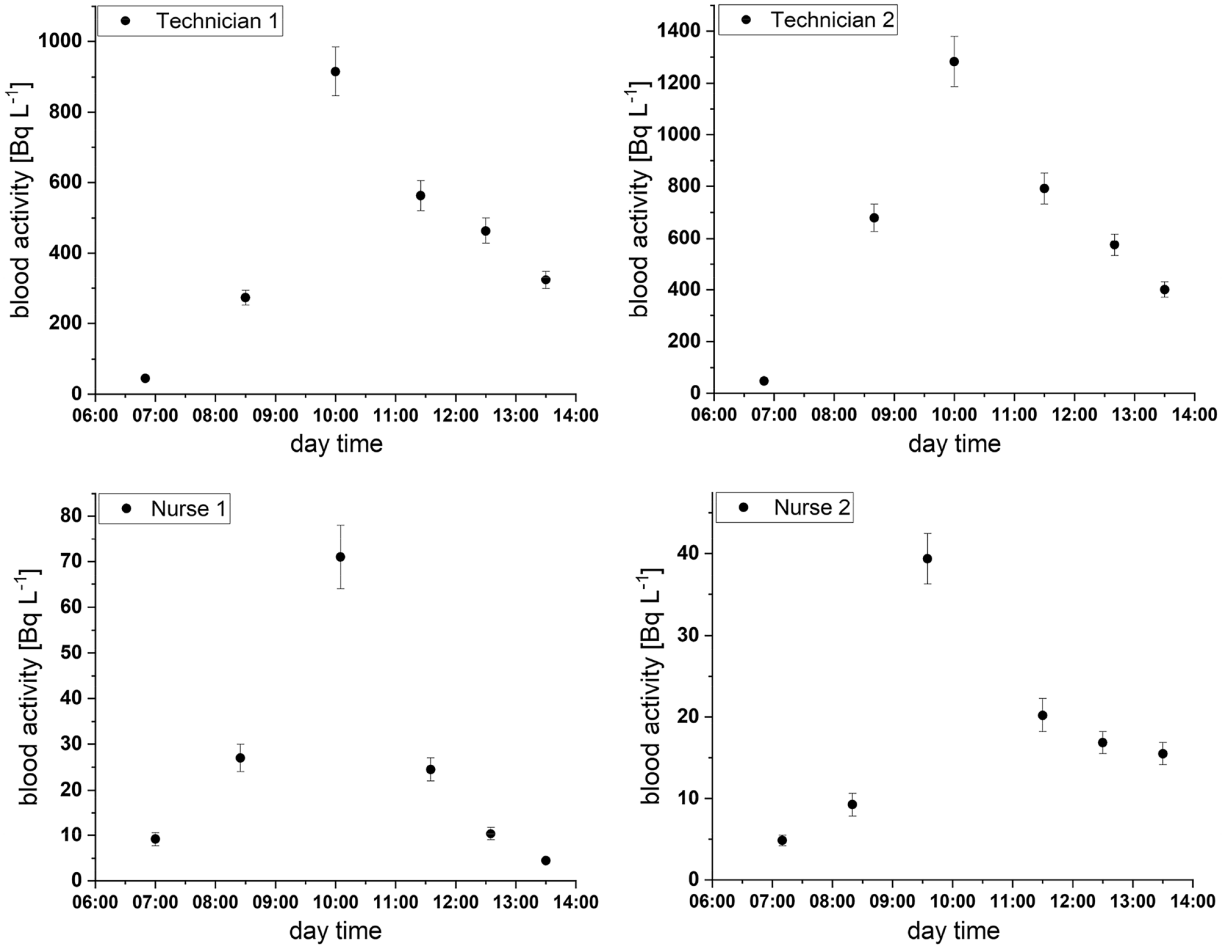
^99m^Tc activity in blood of two technicians (top panels) and two nurses (bottom panels). Error bars represent one sigma measurement error

Additionally ^99m^Tc activity concentration were determined in 24 blood samples and all obtained results were above detection limits. For technicians the maximum ^99m^Tc concentration levels reached 920 ± 70 Bq L^−1^ and 1300 ± 100 Bq L^−1^. For nurses, the estimated activity concentrations were about 10 times lower, namely 71 ± 7 Bq L^−1^ and 39 ± 3 Bq L^−1^. As subsequent procedures of ventilation–perfusion lung scans were performed, the concentration of ^99m^Tc in blood of examined medical personnel was systematically increasing. However, after the last examination, ^99m^Tc was relatively quickly removed. The blood effective half-life time for nuclear medicine personnel was estimated at 2.9 h, 2.4 h, 2.1 h and 0.9 h. Mean effective half-life time was estimated to 2.1 ± 0.8 h. The exact results are shown in Fig. 2.

Applying simple conversion factors and assuming that for every kilogram of body mass there is about 70 mL of blood, it was estimated that the maximum activity in the whole blood of medical personnel was 4600 Bq and 6600 Bq for technicians and 260 Bq and 160 Bq for nurses, respectively.

It is worth noticing that the first blood samples were always collected before starting lung ventilation–perfusion scintigraphy tests. The measured activities which correspond to the background burden and suggest a constant radiation background from ^99m^Tc in the body of medical personnel. Estimated levels were several Bq L^−1^ for technicians and single Bq L^−1^ for nurses. Moreover, medical personnel (especially technicians) had an activity of a several kBq inside their bodies while finishing their working days.

Performed simulations indicated that obtaining such activities in blood of medical personnel was associated with ^99m^Tc activity intake into the respiratory system during a single SPECT scan at levels from 10,000 Bq to 13,500 Bq for technicians and about from 400 to 600 Bq for nurses. This result in daily intakes at the levels ranging from 40,000 Bq up to 54,000 Bq for technicians and from 1600 Bq to 2400 Bq for nurses. These results are in good agreement with the calculated absorptions obtained from air filter measurements.

Assuming intakes during one treatment at the level of 11,000 Bq for technicians and 600 Bq for nurses, the effective dose for one treatment will be 0.17 µSv and 0.01 µSv, respectively. The medical staff performs four treatments on 1 day per week. This corresponds to 200 annual treatments, which results in an annual effective doses of 34 µSv for technicians and 2 µSv for nurses. To exceed by the annual dose limit of 20 mSv (not taking into account the doses from the external radiation fields, which currently oscillate around 1 mSv per year), the technicians would have to perform about 12,000 investigations per year which is clearly impossible.

In the presented study, a relatively small number of samples (12 air samples and 24 blood samples) were measured. Despite this, consistent results were obtained allowing the assessment of the occupational burden. Including more subjects would be difficult due to the high burden associated with the blood sampling, distracting the medical staff from their main responsibility of treating patients.

## Conclusions

This paper presents results of measurements ^99m^Tc activity concentration in air and nuclear medical personnel blood during ventilation–perfusion SPECT lung scan. ^99m^Tc blood activity measurements were conducted at the Nuclear Medicine Department, John Paul II Hospital, Krakow. The results confirm the existence of an internal contamination problem among medical personnel and the need to include them in the radiological protection system. Unfortunately, the vast majority of nuclear medicine facilities around the world do not have systematic solutions for measuring internal contamination among medical personnel. The presented method, based on personal aspirators, is relatively cheap and does not absorb the attention of medical personnel. The method gives promising results and in the future it could find applications in nuclear medicine facilities.

Despite the fact that the estimated intakes appear high, the resulting effective doses are small. However, it is somewhat worrying that as result of long-term internal contamination, a constant radiation background from ^99m^Tc in the body of medical personnel is detectable. It is also a concern that medical personnel finishing their working day may still have a relatively high level of ^99m^Tc activity in their bodies.
